# Intelligent Lecturer Tracking and Capturing System Based on Face Detection and Wireless Sensing Technology

**DOI:** 10.3390/s19194193

**Published:** 2019-09-27

**Authors:** Tan-Hsu Tan, Tien-Ying Kuo, Huibin Liu

**Affiliations:** Department of Electrical Engineering, National Taipei University of Technology, Taipei 10608, Taiwan; thtan@ntut.edu.tw (T.-H.T.); tykuo@ntut.edu.tw (T.-Y.K.)

**Keywords:** lecturer tracking and capturing, face detection, wireless communication, IR thermal sensor

## Abstract

In this paper, we propose an intelligent lecturer tracking and capturing (ILTC) system to automatically record course videos. Real-time and stable lecturer localization is realized by combining face detection with infrared (IR) thermal sensors, preventing detection failure caused by abrupt and rapid movements in face detection and solving the non-real-time sensing problem for IR thermal sensors. Further, the camera is panned automatically by a servo motor controlled with a microcontroller to keep the lecturer in the center of the screen. Experiments were conducted in a classroom and a laboratory. Experimental results demonstrated that the accuracy of the proposed system is much higher than that of the system without IR thermal sensors. The survey of 32 teachers from two universities showed that the proposed system is a more practical utility and meets the demand of increasing online courses.

## 1. Introduction

With the rapid development of education informatization, a wide variety of online courses have received much attention in recent years [1,2]. Most of the top 50 universities in the world, such as Stanford University, Massachusetts Institute of Technology (MIT), and Harvard University, offer online courses on Coursera [2] and edX [3]. This rapid growth makes education, especially higher education, beyond time–space constraints, cross-domain, and borderless. The massive open online course (MOOC) is the most popular type of online course. In 2012, MIT and Harvard University co-invested a free online courses platform called edX. Up to now, edX has offered more than 2500 courses provided by top-ranked universities in the world and industry-leading companies [3]. In the same period, two Stanford university professors, Andrew Ng and Daphne Koller launched Coursera which has opened 3664 courses with 195 partners from 45 countries, according to the official statistics published in July 2019 [2]. These platforms benefit millions of students from all over the world each term, without space–time constraints and borderless.

**Figure 1:**
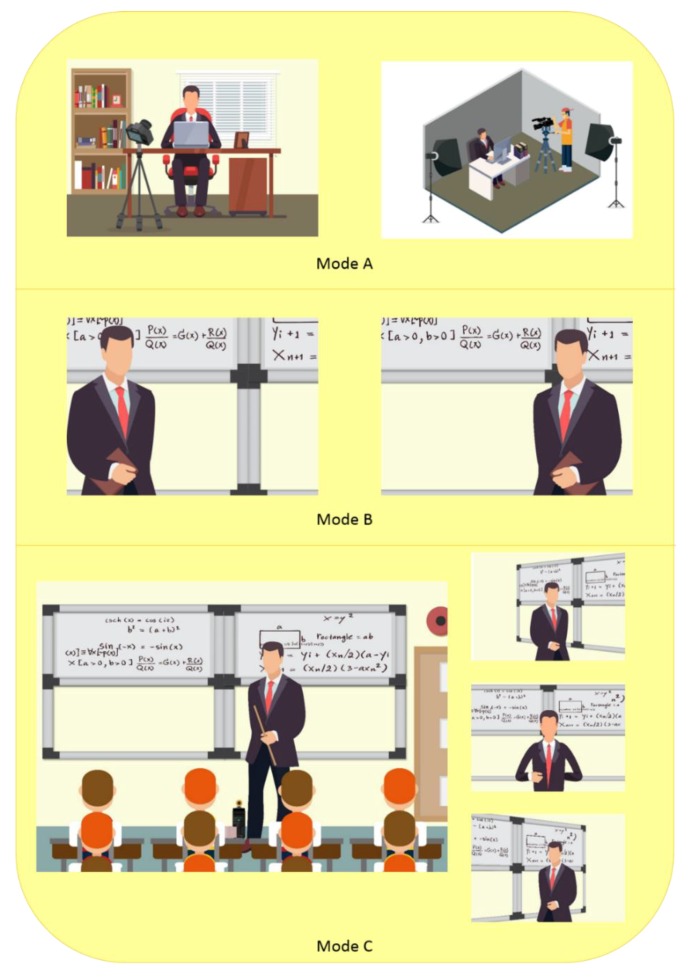
Three modes for recording online course videos. Mode **A**: Capturing in an office or a professional studio. Mode **B**: Capturing with a static camera in a classroom. Mode **C**: Automatic tracking and capturing in a classroom (the proposed system).

However, most of the videos in online course platforms mentioned above are recorded by a technical expert in the professional studios. As described at the top of Figure 1, the videos of MOOCs are usually recorded in studios or offices by photographic specialists using expensive photographic equipment [4,5]. This recording mode not only requires a longer production cycle, but also leads to a higher economic cost [6,7]. Meanwhile, it is hard to excite the lecturer’s enthusiasm without facing any students when the lecture video is recorded in a studio [7]. Certainly, the videos of some online courses are recorded in classrooms directly. For example, the Stanford CS231n course taught by Professor Feifei Li in the Spring of 2017; its videos have been uploaded to YouTube [8]. Nevertheless, the fixed camera limits the scope of the lecturer’s movement as shown in the middle of Figure 1, and the recording process requires manual intervention. Some automatic recording systems are designed to capture the lecturer in a classroom via automatic camera panning and zooming [9], but they cannot be widely applied because of their requirements in specific recording equipment and limitations in frequently changing the focus during the recording operation.

In order to solve the above problems, an intelligent lecturer tracking and capturing (ILTC) system with low cost, stable performance, and convenient construction is proposed in this study, combining artificial intelligence and wireless sensing technology.

There are two functions in the lecturer tracking and capturing system: tracking the lecturer and panning the camera to aim it towards the lecturer as shown in the bottom of Figure 1. First, localization technologies are used to identify the lecturer’s position. Then, the camera is panned automatically to place the lecturer in the center of the screen. By following the above steps, the functions of tracking and capturing are realized.

The existing localization technologies usually use special photographic equipment, such as panoramic cameras [10], PTZ cameras [11], or multiple cameras [12], which require high costs to set up in a classroom and especially to construct a large number of intelligent classrooms. Some classrooms in cram schools have been equipped with an automatic recording system based on contactless sensors, partly having the function of automatic camera panning. However, the rotation angle of camera panning has only a few choices (maybe 2). The more serious problem is that time synchronization amongst the sensors is difficult to achieve, and time-delay occurs when the localization is only based on sensors [13]. Another branch of localization technology is locating the tracked object via portable sensors, such as magnetometers [14], accelerometers, and photodiodes [15]. These methods are also not suitable to track lecturers due to the accompanying inconvenience.

In this study, face detection is combined with wireless sensing technology to realize real-time and stable lecturer localization. After that, a microcontroller, providing communication and control mechanism, and a servo motor with low power are used to pan the camera.

In summary, the main contributions of the ILTC system are threefold:

1. Proposing an intelligent lecturer tracking and capturing system with low-cost, real-time, stable, self-adjusting, and contactless devices.
2. Realizing face detection and capturing by one camera and optimizing the network model with Intel OpenVINO Toolkit to implement the system on CPU in real-time without pre-installing Caffe or TensorFlow.
3. Preventing detection failure caused by abrupt and rapid movements in face detection and solving the non-real-time sensing problem for IR thermal sensors through the combination of face detection and wireless sensing technology.

## 2. Proposed Method

**Figure 2:**
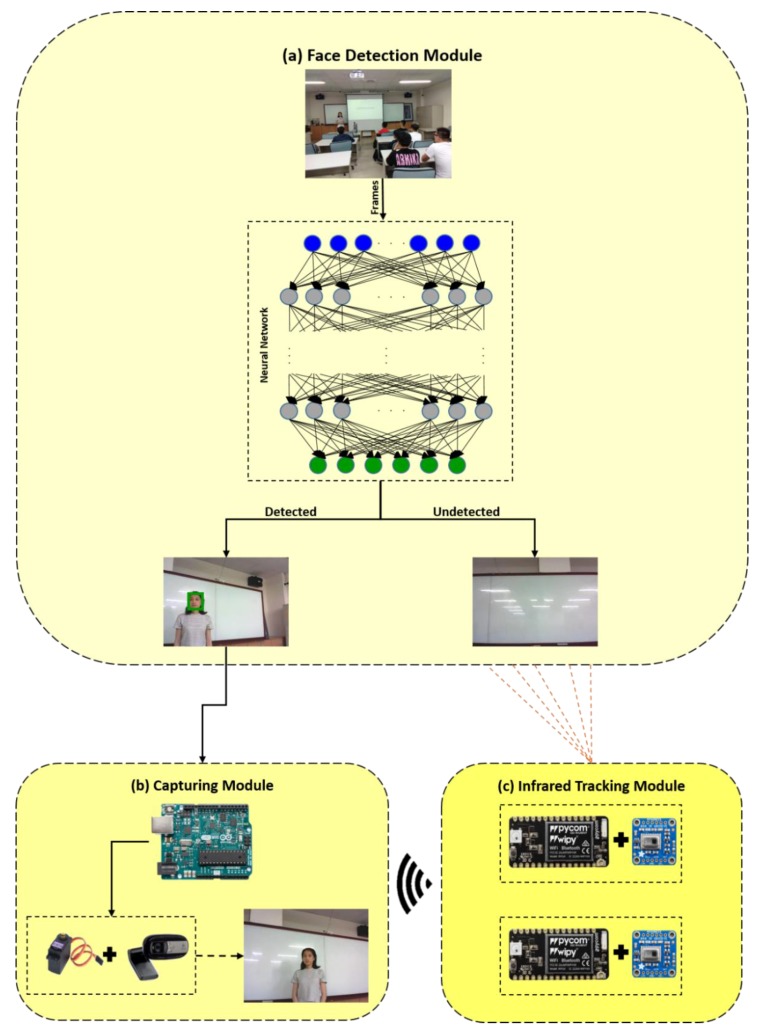
Framework of the intelligent lecturer tracking and capturing (ILTC) system: (**a**) face detection module; (**b**) capturing module; (**c**) infrared tracking module.

In this section, we propose an intelligent lecturer tracking and capturing system, as depicted in Figure 2, comprising the following three modules: (a) face detection module, (b) capturing module, and (c) infrared tracking module. First, face detection is employed to locate the lecturer. If the lecturer is detected in the face detection module, the camera is panned by a servo motor in the capturing module based on the detected result. Otherwise, the infrared tracking module is involved to locate the lecturer via two IR thermal sensors and transmits the location of the lecturer to the capturing module via the wireless communication network. Finally, the lecturer is captured by the camera and kept in the center of the screen during the capturing process.

### 2.1. Face Detection Module

Tracking-by-detection is a popular method for object tracking [16,17]. In the proposed lecturer tracking and capturing system, face detection is used to estimate the location of the lecturer because other body parts of the lecturer may be occluded by the podium. In the past few years, face detection has matured to be used ubiquitously in computer vision systems. Face detection for advanced driving-assistance systems (ADAS) [18] is employed in the face detection module to realize tracking- by-detection in the ILTC system, which is designed for driver monitoring and suitable for lecturer tracking. Both scenarios focus on capturing a person’s face and upper limbs. Furthermore, the optimizer of Intel OpenVINO Toolkit [19] is utilized to improve the computation efficiency of face detection. 

#### 2.1.1. Backbone

Face detection for ADAS is inspired by Single Shot MultiBox Detector (SSD) [20] and MobileNet [21]. The former is a one-stage detection method, which imposes prior boxes to handle different-sized objects on several feature maps with different resolutions and predicts both location and confidences for each prior box. The latter replaces the standard convolution with the depthwise and 1 × 1 pointwise separable convolution [22] to improve the detection speed. 

In the proposed face detection module, MobileNet is introduced to avoid the degradation of the detection speed for SSD on high-resolution input. By introducing depthwise and pointwise convolutional layers, the computational load is greatly reduced. For the standard convolution layer with *H* × *W* input size, *C* channels and *K* 3 × 3 kernals, its computational cost is *H* × *W* × *C* × *K* × 3 × 3. On the contrary, the computational costs of the depthwise and the pointwise separable layer are *H* × *W* × *C* × 3 × 3 and *H* × *W* × *C* × *K* × 1 × 1, respectively. The total computational cost is *H* × *W* × *C* × (3 × 3 + *K*) The compression ratio of the computational costs is calculated by the following equation:

(1) $$R=\frac{H\times W\times C\times \left(3\times 3+K\right)}{H\times W\times C\times K\times 3\times 3}\;=\frac{3\times 3+K}{K\times 3\times 3}$$

**Figure 3:**
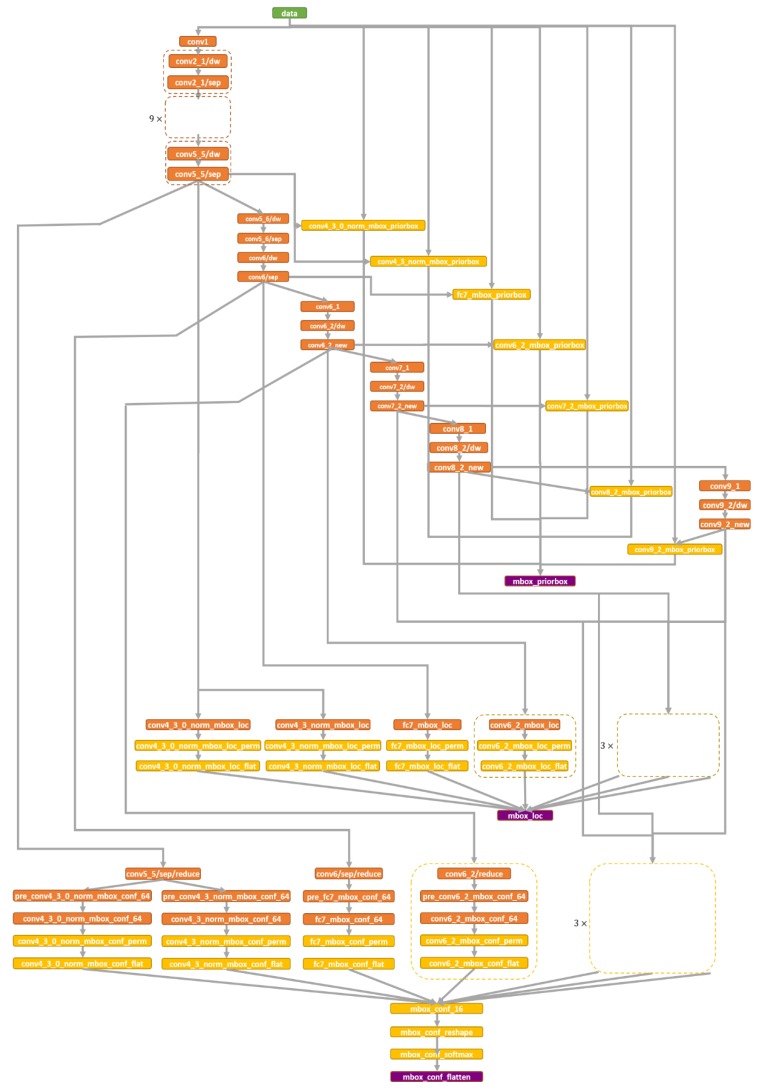
Architecture of the face detection network.

For example, if *K* is 128, *R* is approximately equal to 0.12. The backbone of the face detection module is illustrated in Figure 3. The similar parts in the architecture of the network are condensed and the layers are named according to ref. [18]. Both the depthwise and pointwise convolutional layers are followed by the nonlinear Batchnorm operation and ReLU activation function.

#### 2.1.2. OpenVINO

**Figure 4:**
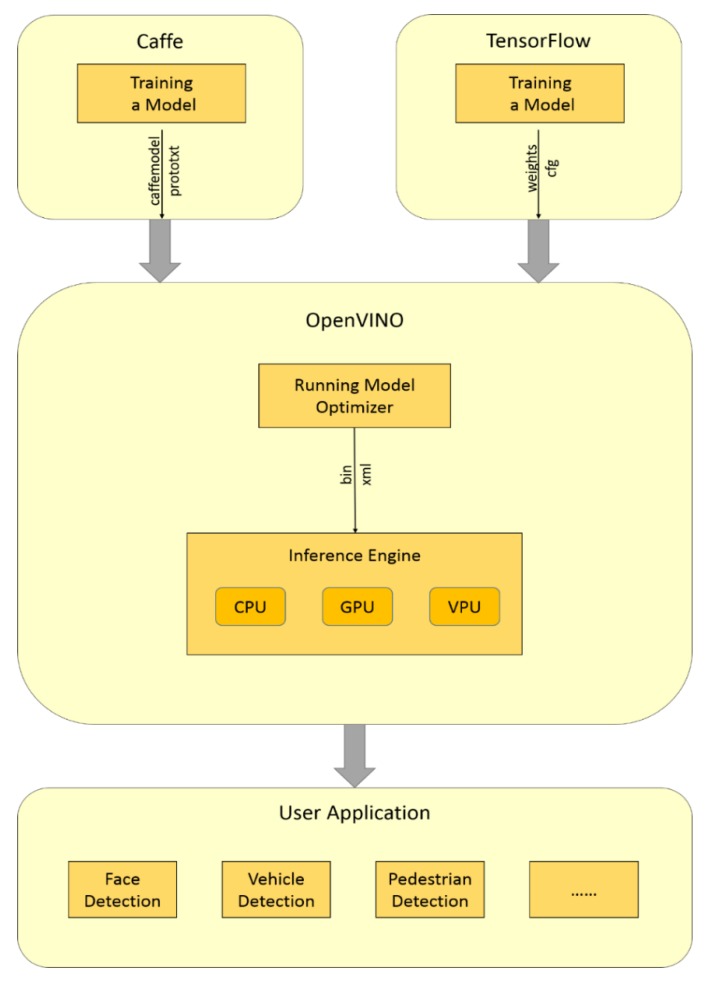
Model optimization with OpenVINO.

OpenVINO is an Intel DL deployment toolkit [19] for quickly accomplishing object detection, action recognition, and other behaviors that emulate human vision, which can accelerate and deploy CNNs on Intel platforms: CPU, GPU, or VPU. As illustrated in Figure 4, the model trained in Caffe or TensorFlow is optimized by OpenVINO. The configuration and weight files of the model are converted to bin and XML files, respectively. For this reason, the CNNs can run on a general-purpose CPU in real-time [23,24].

In the proposed method, the face detection model is optimized by OpenVINO and runs at 25 frames per second on CPU. When the lecturer moves at normal speed, he or she is captured by the camera and is in the center of the screen via the panning of a servo motor to adjust the angle of the camera. The servo motor is controlled by Arduino Uno Wifi [25] and rotates to a certain angle based on the location result of the face detection module, which are described in detail below.

### 2.2. Capturing Module

Arduino Uno WiFi is the most important component in the proposed capturing module, which integrates the microcontroller ATmega328 and WiFi ESP8266 [25]. As depicted in Figure 2, Arduino Uno WiFi provides three functions:

1. Communicating with the computer via the universal serial bus.
2. Controlling the servo motor to rotate the camera mounted on the motor.
3. Receiving data collected by wireless stations, which are connected with IR thermal sensors.

As mentioned in the previous section, the frames captured by the camera are processed in the face detection module to locate the lecturer. Based on the location of the lecturer’s face, the servo motor rotates to a certain angle. The angle is calculated using the following equation:

(2) $$A_{i}=\{\begin{array}{c} A_{i-1}\;\;\left|C_{l}-C\right|\leq T\\ A_{i-1}-\left(C_{l}-C\right)/C\;otherwise \end{array},$$

where *A_i_* and *A_i_*_−1_ are respectively the angles of the servo motor for the current frame and the previous frame. *C* is the center of the frame in the horizontal direction, which is equal to one-half of the width of the frame. *C_l_* is the center of the lecturer in the horizontal direction. *T* is a threshold for rotation. *T* is set to 50 when the resolution of the frame is 450 × 320. Specifically, if the horizontal offset between the lecturer and the screen center is less than 50 pixels, the camera need not be rotated by the servo motor to avoid frequent movement. Meanwhile, the rotation speed is less than 1 degree per frame to make the camera move smoothly. 

### 2.3. Infrared Tracking Module

For smooth motion, the lecturer can be detected in the proposed face detection module. However, if the lecturer moves abruptly or rapidly, the face detection method will fail to track the object. It is a general problem in learning-based tracking methods [26,27]. To overcome this difficulty, IR thermal sensors are introduced to detect the lecturer when the face detection method does not work in the ILTC system. As shown in the lower right part of Figure 2, two AMG8833 IR thermal sensors with a sampling rate of 10 fps are employed to detect the lecturer. Furthermore, two WiFi modules (WiPy 3.0) are connected with the two sensors, respectively, to transmit temperature data to the Arduino Uno WiFi via the wireless communication network. 

#### 2.3.1. IR Thermal Sensors

AMG8833 (GRIDEYE) is an 8 × 8 array of IR thermal sensors, which can measure temperatures ranging from 0 °C to 80 °C and detect a human from a distance of up to 7 m [28,29,30]. The 8-connected component labeling algorithm [31] is used to distinguish the human from noise. If the maximum value in the 8 × 8 array is higher than room temperature, subsequently, it is determined whether the amount of the grids connected to the grid with the maximum temperature is greater than or equal to 2.

Accordingly, it is considered that the lecturer is detected only when the maximum value is higher than room temperature and the corresponding amount of the grid of the connected grids is greater than or equal to 2. In both scenarios, room temperature is always kept below 27 °C using air conditioners. When the maximum value among the 64 grids is higher than 27, the 8-connected component labeling method is introduced to identify the noise. The process of 8-connected component labeling is indicated by the following equation:

(3) $$l_{r,c}=\{\begin{array}{c} 0\;\;\;i_{r,c}=0\\ l_{min\;}\exists \left(i,j\right)\in \left\{\left(r-1,c-1\right),\left(r-1,c\right),\left(r-1,c+1\right),\left(r,c-1\right)\right\},l_{i,j}>0\\ l_{new}\;\;\;otherwise \end{array},$$

For the current pixel (r,c), its label $l_{r,c}$ is set to zero if the intensity of the pixel $i_{r,c}$ is zero. Alternatively, if there exist nonzero values in the upper left, upper, upper right, and left neighbors, $l_{r,c}$ is set to the minimum of the labels in the four neighbors. Otherwise, $l_{r,c}$ is set to a new value which is numbered sequentially. Afterwards, the connected pixels are relabeled with the same number by traversing the image (top-to-bottom, left-to-right). Finally, the total amounts of the connected pixels for each number are counted. In the ILTC system, it need only count the total amount of the connected pixels to the grid with maximum temperature.

The data obtained by AMG8833 sensors include maximum temperature, row, column, and the totality of the connected pixels. The next step is transferring the data to Arduino UNO WiFi in the capturing module via the wireless communication device.

#### 2.3.2. Wireless Communication

**Figure 5:**
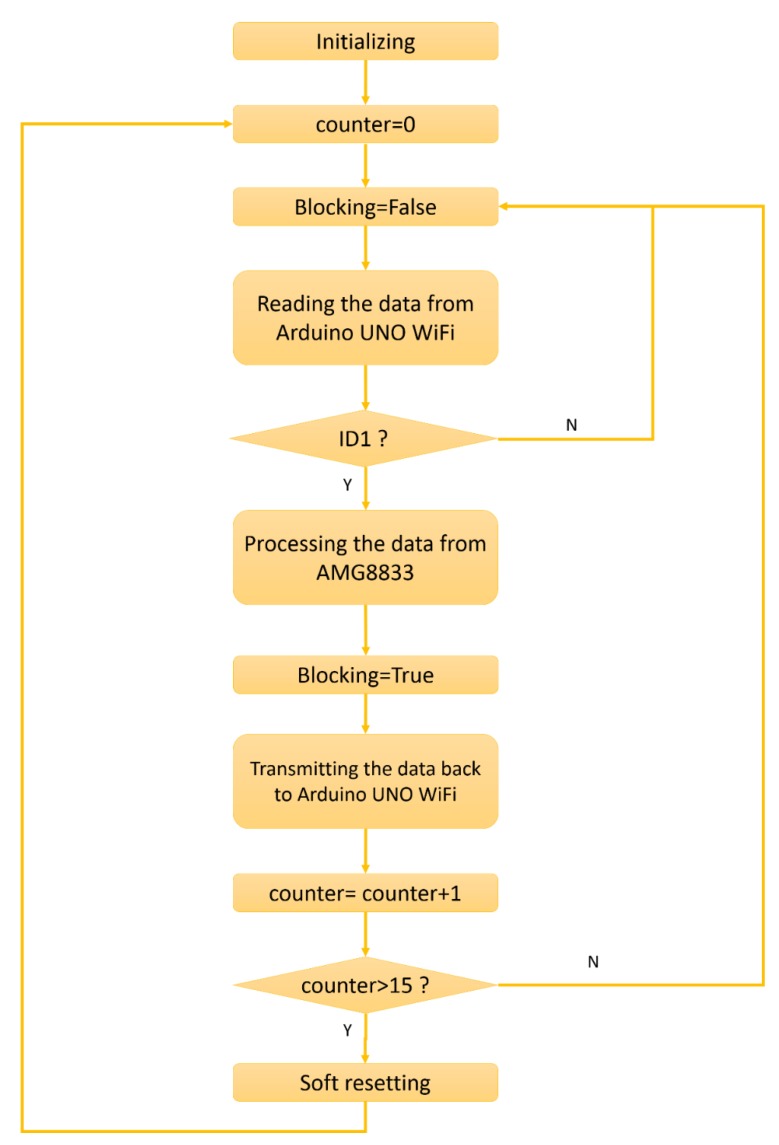
Execution flowchart of WiPy 3.0 named ‘ID1’.

WiPy 3.0 is introduced to realize communication between the AMG8833 and the Arduino UNO WiFi, which is an enabled WiFi and Bluetooth IoT development platform [32]. Two WiPy 3.0 modules are connected to two AMG8833 sensors respectively. Arduino UNO WiFi sends out ‘ID1’ and ‘ID2’ commands alternately via the wireless communication network. Two WiPy 3.0 modules respond to ‘ID1’ and ‘ID2’ respectively. The execution flowchart of WiPy 3.0 named ‘ID1’ is indicated in Figure 5.

First, the wireless network connection is initialized and the counter is set to zero. Then, the data from Arduino UNO WiFi in the capturing module is read via the wireless network. If the data is ‘ID1’, the processed data is transmitted from AMG8833 back to Arduino UNO WiFi. Further, the blocking mode is set to False before reading data from the socket and True before transmitting data to switch between blocking mode and non-blocking mode. The goal here is to avoid blocking up the program and ensure transmitting all pending data. Furthermore, soft resetting is executed to solve the memory shortage problem caused by synchronizing, according to the advice from the official website of Pycom [33].

In summary, the face detection module is responsible for locating the lecturer and the capturing module is responsible for rotating the camera. If the face detection module fails to obtain the location of the lecturer, the infrared tracking module is involved to locate the lecturer via detecting his/her temperature. The rotation angle of the servo motor is determined by the detected results of the face detection module or the infrared tracking module.

## 3. Experimental Results

In this section, the experimental results are presented to evaluate the performance of the proposed ILTC system. The first subsection describes the experimental environment. The qualitative and quantitative analyses are then described. The last subsection shows the survey of 32 teachers on three modes for recording online course videos.

### 3.1. Experimental Environment

**Figure 6:**
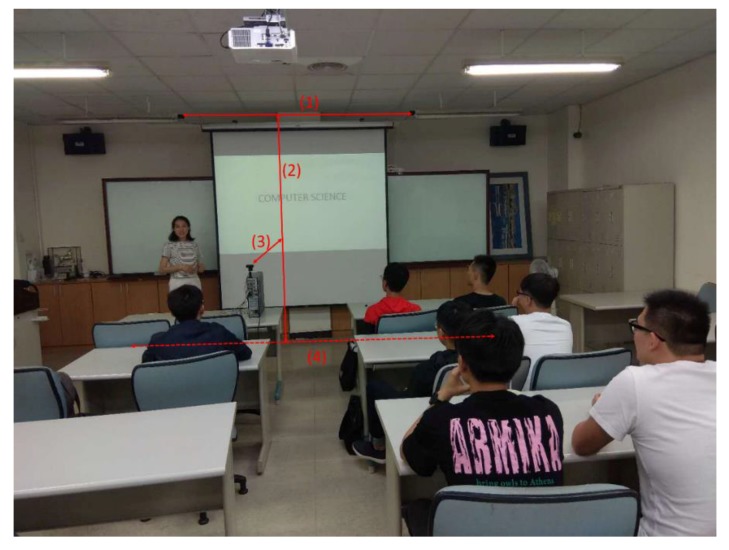
One of the experimental scenarios.

The experiments were carried out in a classroom and a laboratory, respectively, by use of Python 3.6 with a 3.2 GHz Intel i7-8700 CPU and 32 GB RAM. The resolution of the videos is 450 × 320 and the frame rate is 24 fps. Figure 6 shows one of the experimental scenarios, the classroom.

The experimental results are related to four distances, which determine the capturing range. As depicted in Figure 6, they are (1) the distance between two AMG8833 sensors, (2) the distance between the sensor and the ground, (3) the distance between the camera and the whiteboard and (4) the range of the lecturer’s movement. Table 1 gives the values of the distances in the classroom and the laboratory.

**Figure 7:**
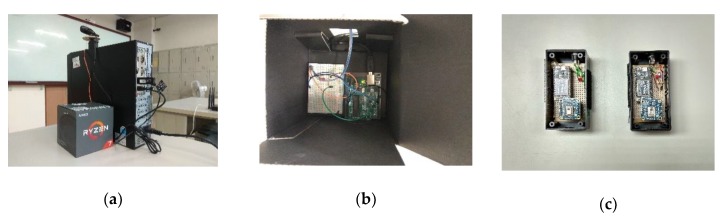
Devices used in experiments: (**a**) Computer placed in the first row; (**b**) Arduino UNO WiFi connected with the computer; (**c**) AMG8833 integrated with WiPy 3.0.

As shown in Figure 7a, a computer was placed in the first row of the classroom, connected with Arduino UNO WiFi, shown in Figure 7b. Figure 7c presents AMG8833 integrated with WiPy 3.0. Table 2 shows the list of hardware and software platforms used in the ILTC system.

### 3.2. Qualitative Analysis

The key technologies of the ILTC system are human localization and tracking. Accordingly, we compared the pros and cons of the proposed system with the existing localization and tracking methods, as described in Table 3. There are two typical categories of the existing methods: device-based [10,12,13,14,15] and learning-based methods [26,27]. Some of the device-based methods require special and expensive devices. Further, most of them are the contact method and the non-real-time system. For state-of-the-art learning-based human localization and tracking methods, GPU is essential to achieve high accuracy and performance. Furthermore, abrupt and rapid movements cause tracking failures in the learning-based methods.

Table 3 shows that the proposed method is the optimal scheme for lecturer tracking and capturing system in the compared methods, benefiting from low cost, real-time stable performance, contactless devices, and convenient construction. However, although switching between blocking and non-blocking modes and soft resetting were introduced to improve the stability of the ILTC system, the temporary detecting failure still occurred because of the limitations of the sensor performance. That is to say, the tracked object is lost temporarily. This may be due to two reasons: the low sampling rate of AMG8833 IR thermal sensors and the time-consuming procedure for soft resetting of the sensors to free up the memory. This topic is worth pursuing in future work.

### 3.3. Quantitative Analysis 

To evaluate the accuracy of the ILTC system, Center_rate, the ratio of the number of the frames, in which the lecturer is in the center of the screen, and the number of all frames is calculated based on the following equation:

(4) Center_rate=Center_numFrame_num,

where Center_num is the number of the frames, in which the lecturer is captured in the center of the screen. According to Section 2.2, if the horizontal offset between the lecturer and the screen center is less than 50 pixels, it implies that the lecturer is in the center of the screen. Frame_num is the number of all frames in the video. When the lecturer moves frequently, there are lots of frames captured by the panning camera, so another ratio is considered as below.

(5) In_rate=In_numFrame_num,

In_num is the number of the frames in which the lecturer appears on the screen. To illustrate the role of IR thermal sensors, the ratios are recalculated after removing the AMG8833 sensors from the ILTC system. Ten videos are recorded by the system without AMG8833 sensors. To compare with them, another ten videos are further recorded by the entire ILTC system. Table 4 gives the comparative result of the ratios in the two cases.

As indicated in Table 4, Center_rate in the entire system varies between 55.72% and 66.02%. In_rate in the entire system varies between 83.05% and 92.14%. However, the values of these items in the system without AMG8833 sensors are much lower than the values in the entire system. As shown in the last row of Table 4, the average of Center_rate of the entire system is greater than that of the system without AMG8833 sensors by 15.61%, and its In_rate average is 21.40% greater. Consequently, the introduction of IR thermal sensors greatly improves the accuracy of the ILTC system. 

A total of 20 videos are recorded by the entire ILTC system in two scenarios. The results of twenty videos are depicted in Table 5. The first seven videos are captured in the laboratory, and others are captured in the classroom. The average Center_rate and In_rate of the videos captured in two scenarios are calculated respectively. The total average of 20 videos is also calculated and shown in the last row of Table 5.

As shown in Table 5, Center_rate of the videos captured in the laboratory varies between 55.72% and 68.38%. In_rate varies between 83.05% and 91.10%. Center_rate of the videos captured in the classroom varies between 58.28% and 72.79%. In_rate varies between 84.09% and 93.90%. The average Center_rate of the videos recorded in the classroom is 2.26% greater than the laboratory, and the average In_rate is 1.68% greater. In theory, the shorter the distance between the sensor and the ground, the higher the accuracy of the ILTC system. Taken altogether, seven out of twenty videos have Center_rate greater than 65% and six out of them have In_rate greater than 90%. The average Center_rate is 63.97%, and the average In_rate is 88.20%.

### 3.4. Survey

In order to assess the practicality of the ILTC system, we surveyed 32 teachers from two universities including departments of mechanics, chemistry, electronics, management, and general education on three modes for recording online course videos described in Figure 1, which are:

1. Mode A: Capturing in office or professional studio.
2. Mode B: Capturing with static camera in classroom.
3. Mode C: Auto tracking and capturing in classroom.

The 32 teachers scored the four items of the three modes described on the survey questionnaire, using a 5-point Likert scale (1=strongly disagree, 5=strongly agree). The surveyed items include:

1. Acceptability: reasonable time and financial costs.
2. Simplicity: simple to operate, non-manually.
3. Appeal: helpful to bring out the enthusiasm of the lecturer.
4. Effectiveness: effective to satisfy the audience’s requirement.

**Figure 8:**
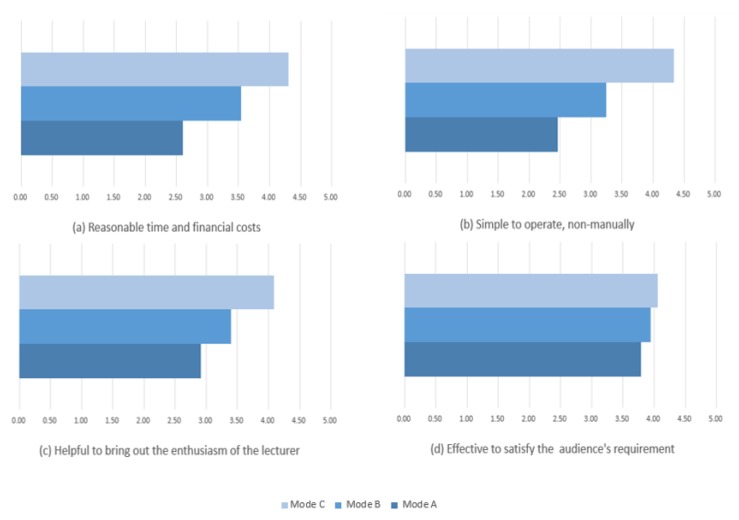
Survey on the three modes for recording online course videos.

For easy comparison between the three modes, the mean score is calculated on each item. As shown in Figure 8, all of the scores in mode C are greater than those of the other two modes, especially in terms of acceptability and simplicity. That is to say, most of the respondents considered that mode C has more reasonable costs than the other two modes. Meanwhile, they thought that Mode C was the simplest way to operate the capturing system among the compared modes. All modes were considered effective to satisfy the audience’s requirement. Of the survey respondents 37.5% had experience in recording course video in professional studios. All of them gave the lowest scores in acceptability to mode A for its high time and financial costs. 

## 4. Conclusions

This study proposes an intelligent lecturer tracking and capturing system by taking the advantages of artificial intelligence and wireless sensing technology. The proposed ILTC system consists of three modules: face detection module, capturing module, and infrared tracking module. In the face detection module, the location of the lecturer is obtained by detecting the face of the lecturer. Afterwards, the camera is panned toward the lecturer in the capturing module. If the face detection module fails to detect the lecturer, an infrared tracking module can contribute to lecturer detection by IR thermal sensors. The experimental result indicates that the proposed system can automatically track and capture the lecturer, placing him/her in the center of the screen. By comparing the proposed system with the other two capturing modes on surveying the teachers from different departments, it was proved that the ILTC system has greater practicality. 

In order to further improve the accuracy of the ILTC system, learning-based tracking methods for abrupt and rapid movements and sensing technologies with low memory requirements can be considered as a topic for future research.

## Figures and Tables

**Table 1:** The distances in the classroom and the laboratory.

Scenario	(1)	(2)	(3)	(4)
Classroom	2.85 m	2.69 m	1.50 m	3.60 m
Laboratory	2.02 m	3.30 m	1.52 m	2.45 m

**Table 2:** Hardware and software platforms used in the intelligent lecturer tracking and capturing (ILTC) system.

Hardware	Software
Arduino UNO WiFi	Arduino IDE 1.8.9
Servo Motor MG996R	Pycharm in Python 3.6
Logitech C170 webcamera	
Adafruit AMG8833 IR thermal sensor	ATOM 1.37.0
Pycom WiPy 3.0	

**Table 3:** Comparative Analysis among the existing localization and tracking methods.

Technology	Pros	Cons
Panoramic camera and WiFi [10]	Convenient construction and low cost	Distorted images, not suitable for great varying illumination and blurred face
Multi cameras [12]	Indoor and outdoor localizations under different time and weather condition	Selected places, multi cameras, contact devices and non-real-time system
Ultra wide band [13]	More robust time-delay localization	Contact devices and non-real-time system
Magnetic field and WiFi [14]	Convenient construction and high accuracy	Contact devices in a fixed body position
Accelerometer and optical receivers [15]	High accuracy	Sensitive to light noise, contact devices
Multi-domain convolutional neural networks [26]	Fast and accurate	GPU-only, fail to track object with abrupt or rapid movement
deep reinforcement learning [27]	Semi-supervised learning and high accuracy	15 fps on GPU, fail to track object with abrupt or rapid movement
Camera, WiFi and IR thermal sensors (the proposed ILTC System)	Low cost, real-time stable performance, contactless devices and convenient construction	Temporary detecting failure

**Table 4:** Comparative result of the ratios in the two cases.

Video	Entire System			Without AMG8833		
	Frame_Num	Center_Rate (%)	In_Rate (%)	Frame_Num	Center_Rate (%)	In_Rate (%)
Video1	1705	55.72	83.28	1124	43.68	69.13
Video2	2405	60.50	91.10	1215	41.07	71.77
Video3	1928	59.02	85.53	1531	53.23	66.04
Video4	1945	66.02	89.97	1693	52.22	66.69
Video5	1999	63.08	86.99	2234	46.20	65.76
Video6	2259	64.81	83.05	1978	49.80	66.73
Video7	2181	58.28	84.09	2089	41.31	61.51
Video8	2405	60.29	87.03	1355	48.63	65.17
Video9	2086	65.00	92.14	1475	45.36	63.73
Video10	2401	63.81	86.30	1666	38.90	59.00
Average	2131	**61.65**	**86.95**	1636	46.04	65.55

**Table 5:** The results of twenty videos captured in two scenarios.

**Video Captured in the Laboratory**	**Center_Num**	**In_Num**	**Frame_Num**	**Center_Rate (%)**	**In_Rate (%)**
Video1	950	1420	1705	55.72	83.28
Video2	1455	2191	2405	60.50	91.10
Video3	1138	1649	1928	59.02	85.53
Video4	1284	1750	1945	66.02	89.97
Video5	1261	1739	1999	63.08	86.99
Video6	1464	1876	2259	64.81	83.05
Video11	1546	2030	2261	68.38	89.78
Average	1300	1808	2072	62.50	87.10
**Video Captured in the Classroom**	**Center_Num**	**In_Num**	**Frame_Num**	**Center_Rate (%)**	**In_Rate (%)**
Video7	1271	1834	2181	58.28	84.09
Video8	1450	2093	2405	60.29	87.03
Video9	1356	1922	2086	65.00	92.14
Video10	1532	2072	2401	63.81	86.30
Video12	2067	2773	2953	70.00	93.90
Video13	2096	2742	3190	65.71	85.96
Video14	1857	2460	2716	68.37	90.57
Video15	2231	2784	3065	72.79	90.83
Video16	1880	2482	2834	66.34	87.58
Video17	1657	2446	2762	59.99	88.56
Video18	2953	4060	4614	64.00	87.99
Video19	2421	3679	3965	61.06	92.79
Video20	2800	3651	4223	66.30	86.46
Average	1967	2692	3030	**64.76**	**88.78**
Total Average	1733	2383	2695	63.97	88.20
